# Supplementary material for: The genetic heterogeneity and drug resistance mechanisms of relapsed refractory multiple myeloma
Source: Nat Commun. 2022 Jun 29;13:3750. doi: 10.1038/s41467-022-31430-0 (PMC9243087; doi:10.1038/s41467-022-31430-0)
Supplement: Supplementary file 1 — Supplementary Information [file 41467_2022_31430_MOESM1_ESM.pdf]

The Genetic Heterogeneity and Drug Resistance Mechanisms of Relapsed Refractory Multiple Myeloma

SUPPLEMENTARY INFORMATION

Supplementary Figs. 1-9

Supplementary Fig. 1

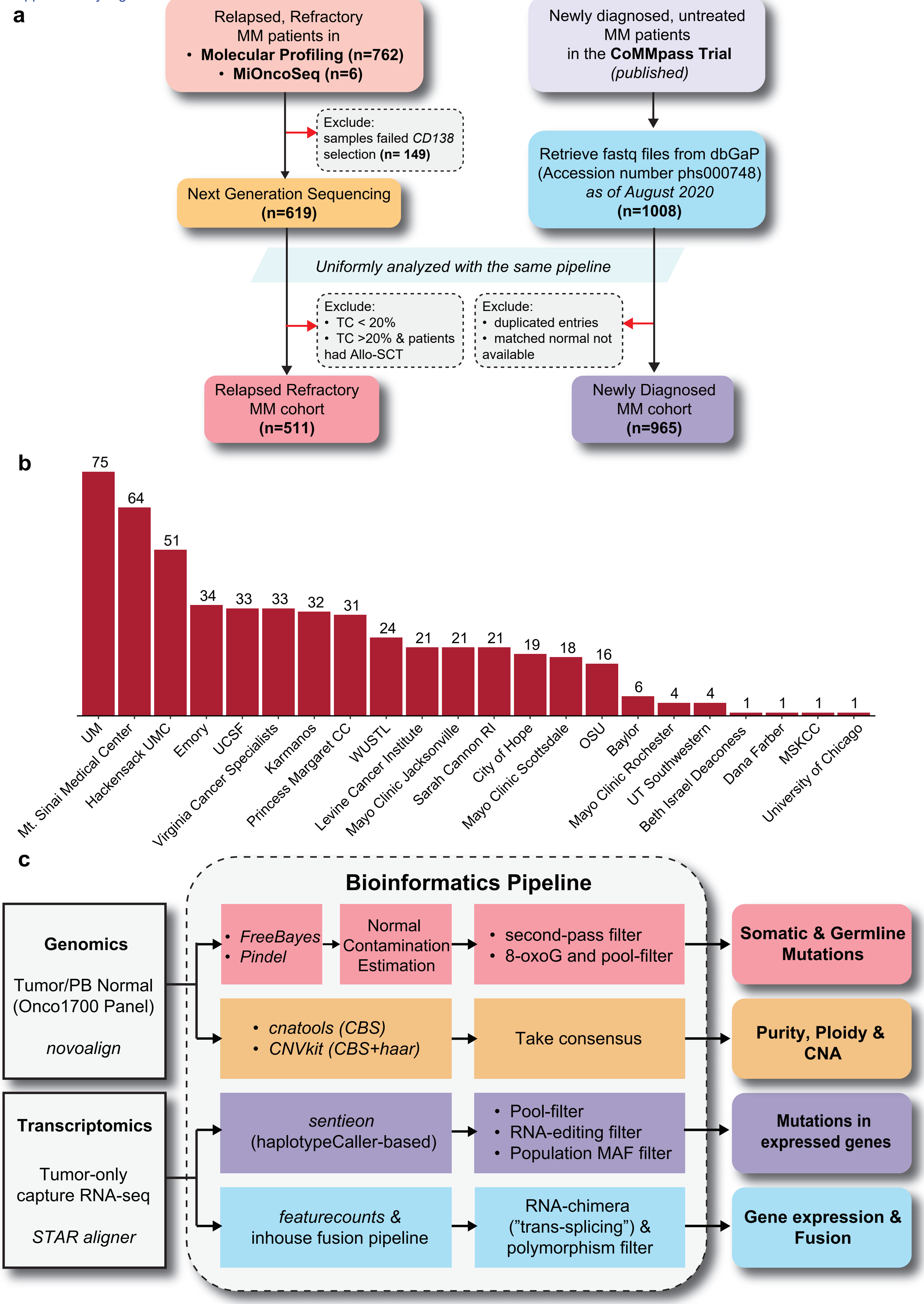

Supplementary Fig. 1 Overview of MMRF Molecular Profiling Initiative cohort and bioinformatics pipeline. a, Schematic depicting the inclusion and exclusion criteria for the relapsed refractory multiple myeloma (RRMM) and newly diagnosed MM (NDMM) cohorts. **b,** Distribution of clinical sites. UM, University of Michigan; UCSF, University of California-San Francisco; WUSTL, Washington University in St. Louis; OSU, Ohio State University; MSKCC, Memorial Sloan Kettering Cancer Center. **c,** Overview of our integrative bioinformatics pipeline (Methods). PB, peripheral blood; CNA, copy-number alterations.

Supplementary Fig. 2

a

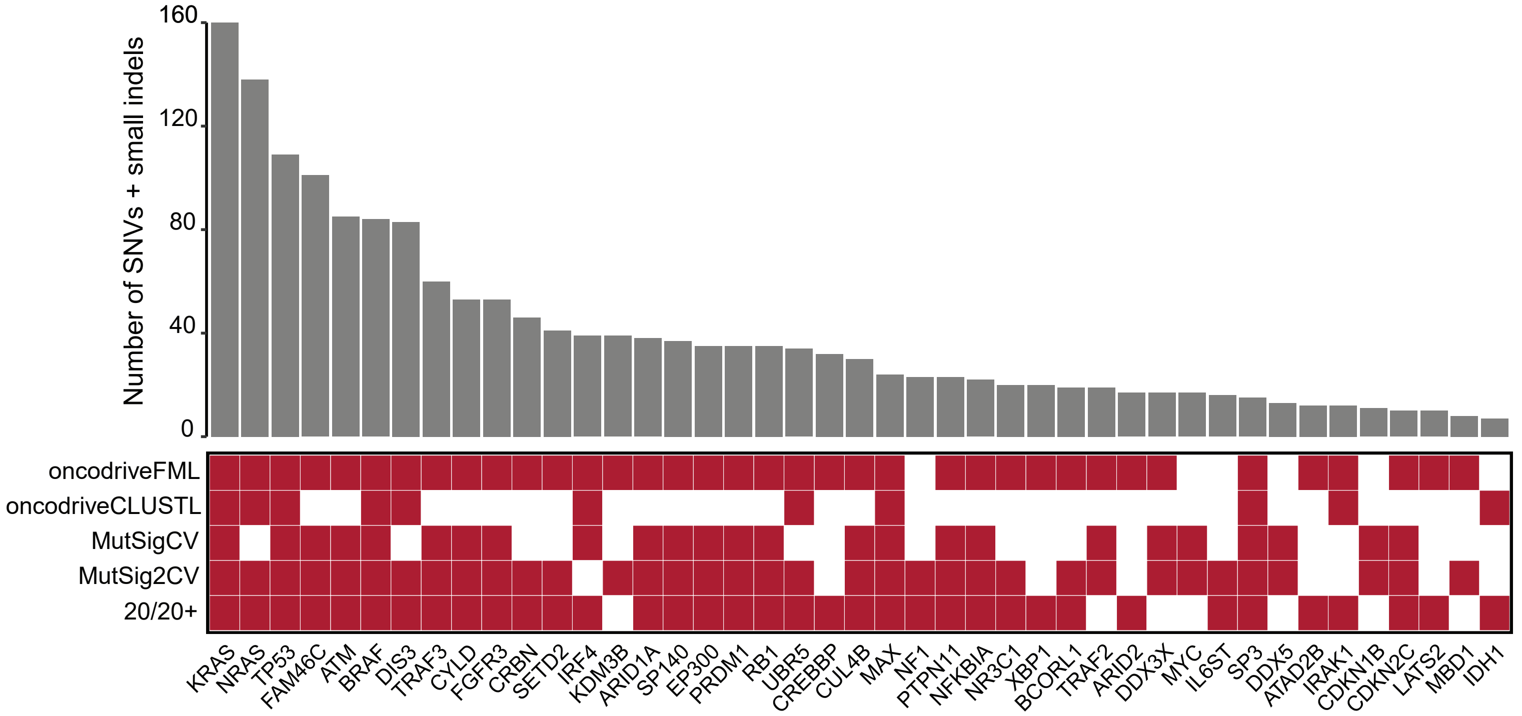

b

Pathways/Cellular Processes	Genes
RAS-RAF Pathway	*KRAS, NRAS, BRAF, PTPN11, NF1, IL6ST*
NF-κB Pathway	*TRAF3, TRAF2, CYLD, NFKBIA, IRAK1*
MYC Pathway	*MYC, MAX, EP300, CREBBP, SP3*
Cell Cycle & DNA Damage Checkpoint	*TP53, RB1, CDKN2C, CKDN1B, ATM, FGFR3, LATS2*
RNA Processing & Maturation	*DIS3, FAM46C, DDX3X, DDX5*
Epigenetics Modifiers/ Transcriptional Coactivators and Corepressors	*KDM3B, SETD2, ARID1A, ARID2, MBD1, IDH1, BCORL1, ATAD2B*
B-Cell Lineage	*PRDM1, SP140, UBR5, IRF4*
Drug Resistance	*CRBN, CUL4B, NR3C1*

c

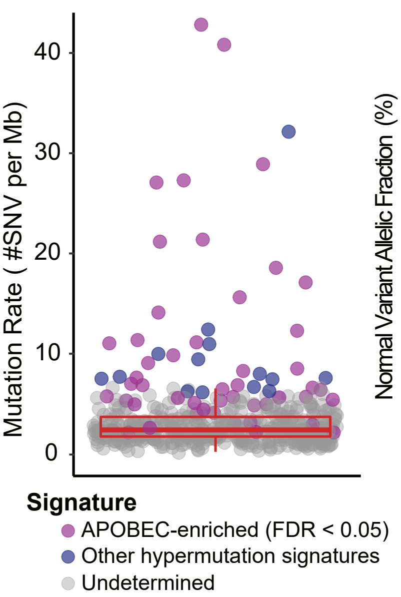

d

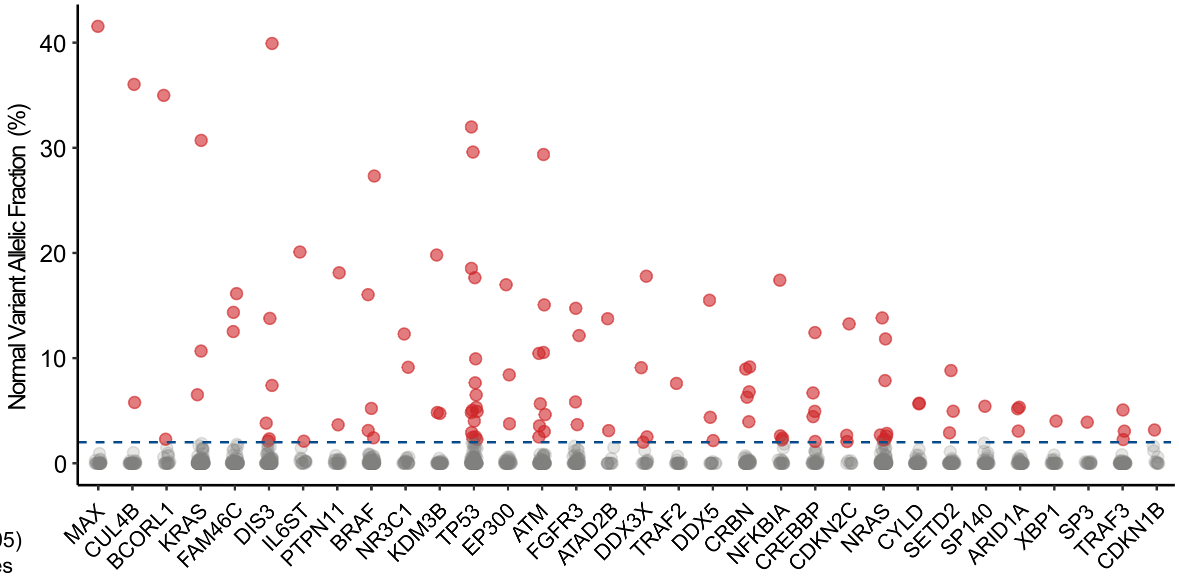

Supplementary Fig. 2 Somatic mutations in RRMM. a, Bottom matrix, genes that were predicted to be significantly mutated by indicated statistical tools (Methods). Top histogram, total number of single nucleotide variant (SNV) and small indels per gene observed in the entire cohort. **b,** Summary of pathways and cellular processes affected by mutations. **c,** Mutation rate (number of point mutations per megabases) in RRMM. APOBEC-enriched and non-APOBEC-enriched cases are highlighted in purple and dark blue. **d,** Most of the significantly mutated genes in our panel were affected by mutations that could also be detected in matched peripheral blood controls with a wide range of variant allelic fractions (VAF) due to the presence of circulating tumor cells (CTCs). Without a CTC-aware filtering strategy, many CTC-associated mutations (in red) would have been missed if an arbitrary cut-off at low VAF (horizontal dashed line) was applied to the normal sample.

Supplementary Fig. 3

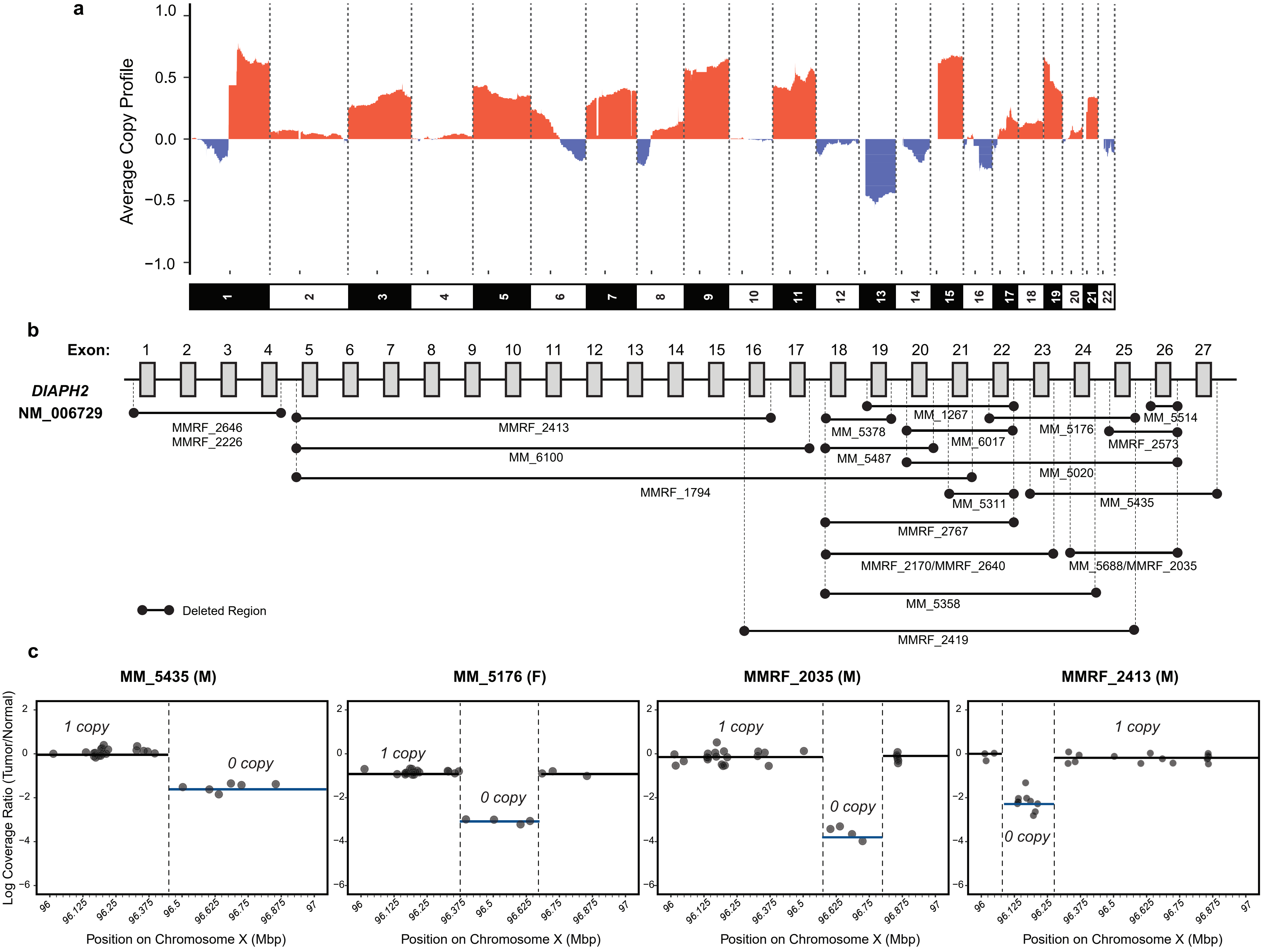

Supplementary Fig. 3 Copy-number alterations in RRMM. a, Average copy-number profile of RRMM. IgH, IgL, IgK, and TCR loci were excluded, as these loci are heavily rearranged and deleted in MM. **b,** *DIAPH2* was frequently affected by focal deletions affecting a single exon or a group of exons in both relapsed refractory (MM_*) and newly diagnosed (MMRF_*) patients. **c,** *DIAPH2* focal losses were homozygous and affected both males (M) and females (F). MM_5176 was an example of a female MM patient with one copy deletion of the entire X chromosome and focal deletion of *DIAPH2* on the remaining allele.

Supplementary Fig. 4

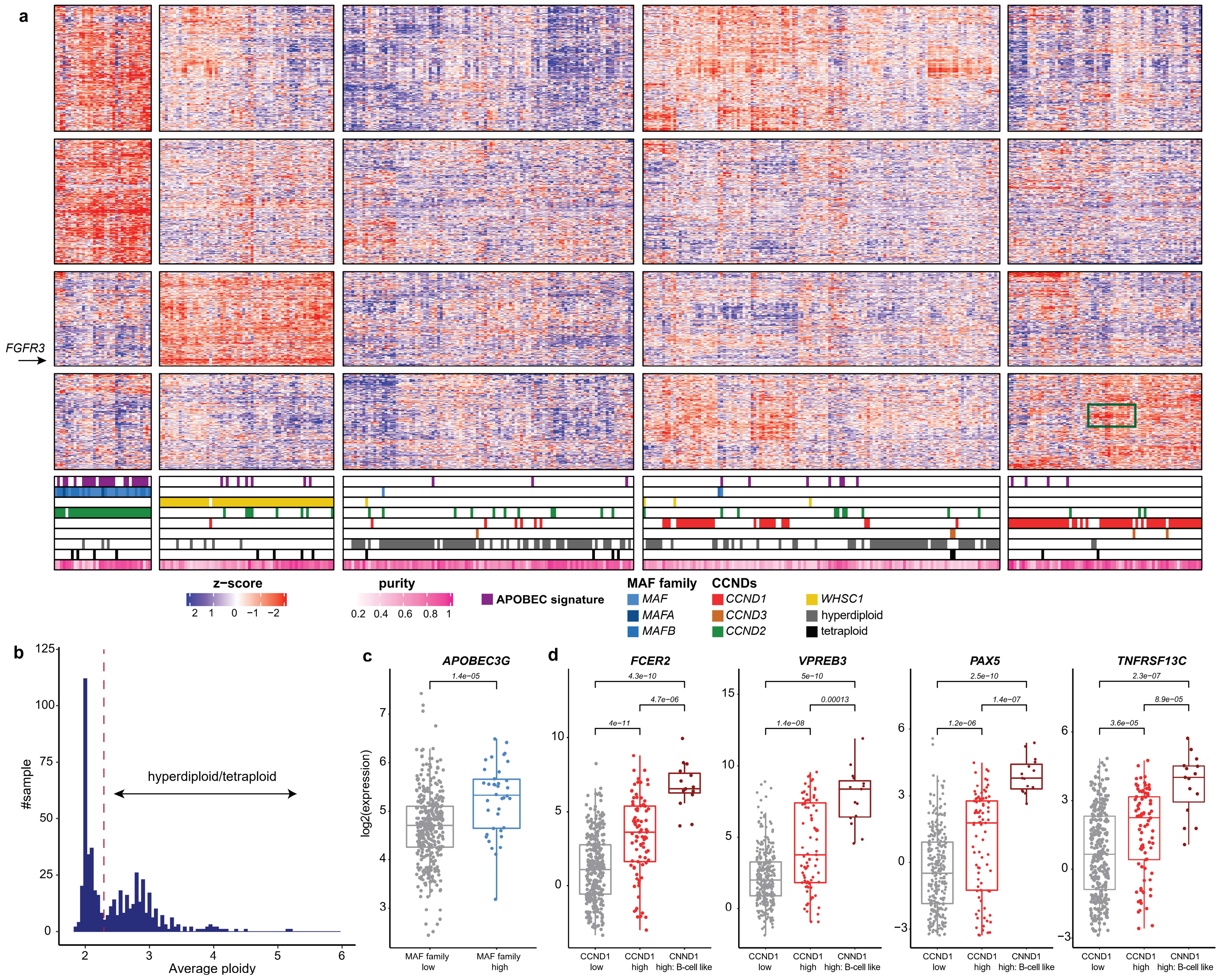

Supplementary Fig. 4 Integrative analyses of mutational signatures and transcriptomic analyses of RRMM. a, k-mean clustering of variance stabilizing transformation (vst) of RNA-seq read-count data (402 samples x 837 genes). For this analysis, samples with tumor purity < 30% and outliers assessed from Principal Component Analyses (PCA) were removed to reduce the effect of contamination from non-plasma cells. Differential expression analyses with edgeR were carried out for cases with top 10% expression of *WHSC1*, *MAF*, and *CCND1* each versus the rest, and genes with FDR < 0.01 and fold-change ≥ 1.5 were included. For the annotation tracks (bottom tracks of the heatmap), the following thresholds were applied: 50 FPKM for *WHSC1*, 50 FPKM for *MAF*, 150 FPKM for *CCND1*. Rare translocations such as *CCND3*, *MAFA,* and *MAFB* were identified using OUTRIDER (Methods). Samples with high-level expression of the *MAF* family genes, *WHSC1*, and cyclin D genes showed distinct transcriptional patterns. *FGFR3* was also upregulated in some *WHSC1*-translocated cases. Samples with high-level expression of the *MAF* family genes associate with APOBEC mutational signatures. **b,** A ploidy score of each sample was calculated as the average copy-number of chromosomes or chromosome arms that are frequently affected by a hyperdiploid phenotype. The distribution of ploidy score was bimodal. A cut-off of 2.3 was used to distinguish diploid from hyperdiploid samples. Samples with a ploidy score ≥ 4 are marked as tetraploid (whole-genome duplication). In the heatmap in **a,** hyperdiploid cases and cases with high expression of translocated partners tended to not overlap. **c,** Samples with high-level expression of the *MAF* family genes (n=38) also had elevated expression of *APOBEC3G* compared to the rest of the cohort (n= 364). **d**, A group of samples clustered in the *CCND1* group (n=15, also highlighted in the green square in the heatmap in **a**) expressed high levels of pre-B cell markers, such as *FCER2* (*CD23*), *VPREB3, PAX5,* and *TNFRSF13C* compared to other *CCND1*-high samples (n=85) and the rest of the cohort (n=302). P-values were calculated using one-sided Wilcoxon rank-sum test. All box plots include the central line as median, box limits as the upper and lower quartiles, and whiskers extend to 1.5 times the interquartile range.

Supplementary Fig. 5

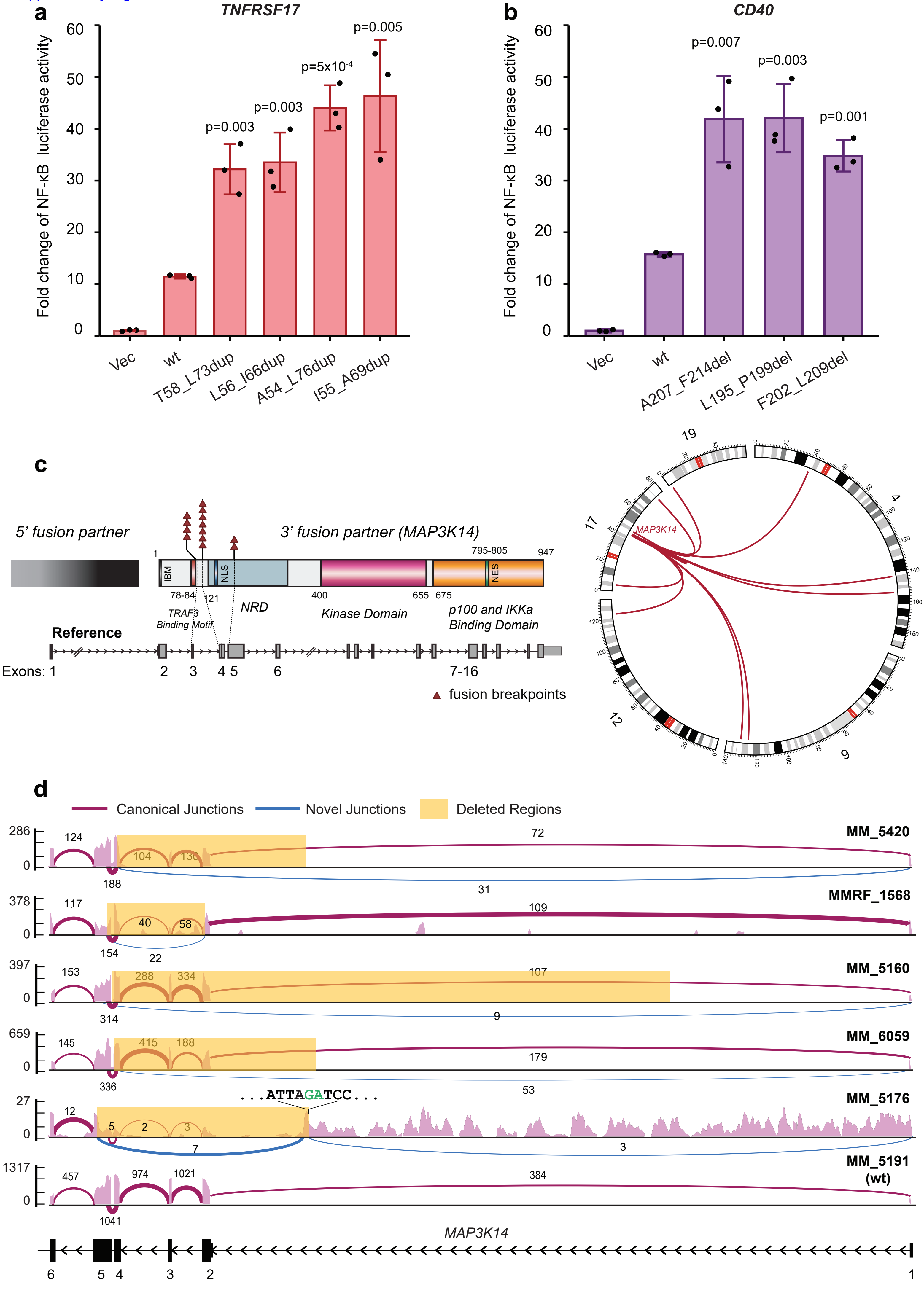

Supplementary Fig. 5 Alterations in the NF-κB pathway. a, b, Fold-change of luciferase reporter activities (relative to the average of control vector) of in-frame insertions in *TNFRSF17* and in-frame deletions in *CD40* in three (n=3) independent experiments. Error bars represent standard deviations. P-values were calculated using one-sided t-test on log_2 fold-change compared to wild-type (wt). Source data are provided as a Source Data file. **c,** Left, schematic for N-terminal fusions of *MAP3K14*. These fusions maintained an intact kinase domain while removing the *TRAF3* binding motif. Right, circos plot depicting the location of *MAP3K14* fusion partners. **d,** Sashimi plot depicts the de novo splice junction observed in cases with *MAP3K14* N-terminal deletions. In MM_5176, there was a de novo splice acceptor (highlighted in green) upstream of the deletion breakpoint in intron 1.

Supplementary Fig. 6

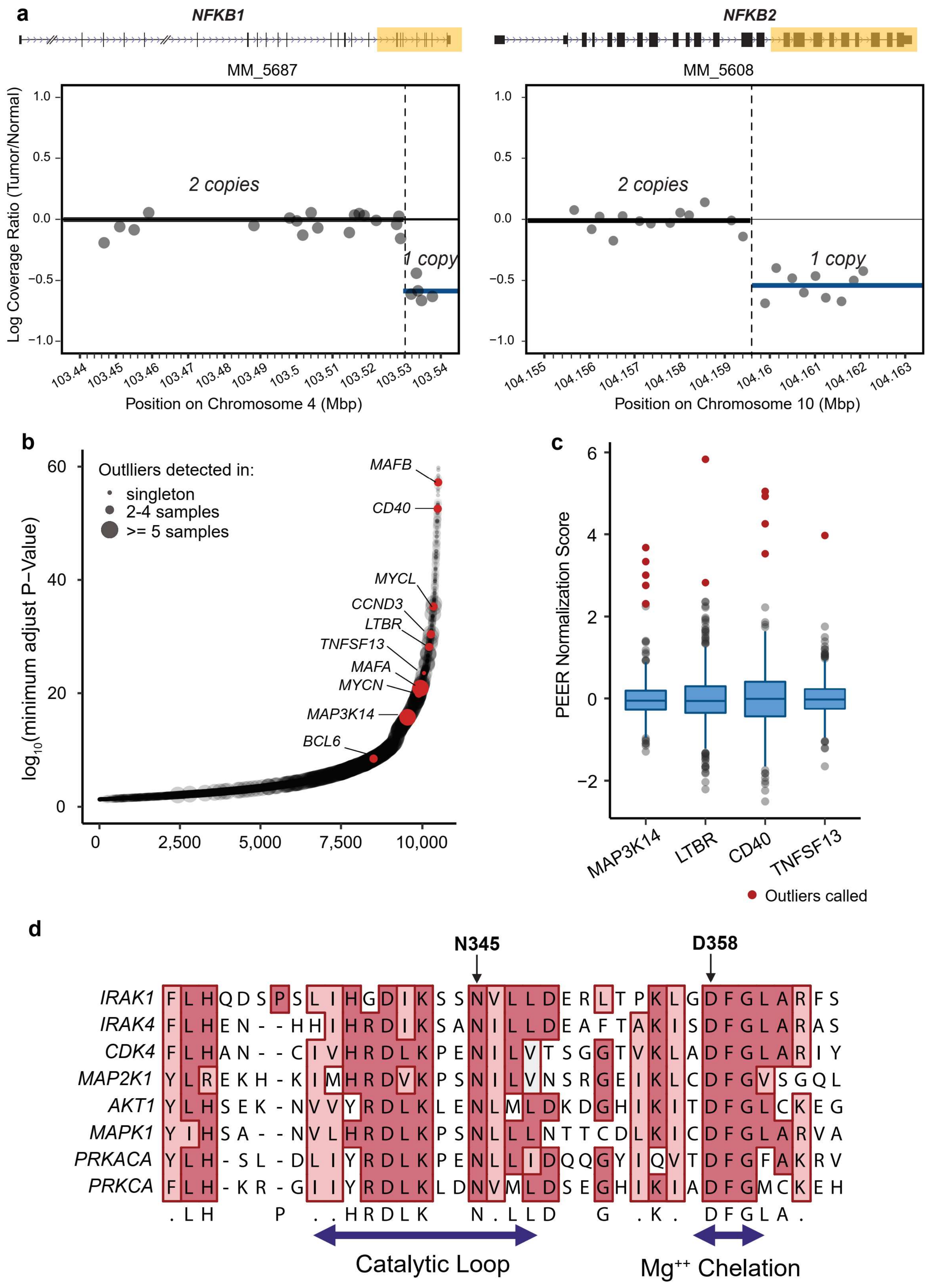

Supplementary Fig. 6 Alterations in the NF-κB pathway (continued). a, Example of cases with C-terminal deletion of *NFKB1* and *NFKB2*. **b,** Prioritization of genes with rare outlier expression. Using OUTRIDER (Methods), the significance of each outlier in each sample was calculated. The minimum P-value of the outlier observed in each gene was used to prioritize the genes. Genes in the NF-κB pathway and MYC pathway are indicated. **c,** Boxplot of expression values normalized using PEER (Methods) for *MAP3K14*, *LTBR*, *CD40,* and *TNFSF13* for the same set of samples included in integrative transcriptomic analyses as explained in **Supplementary Figure 4** (n=402). All box plots include the central line as median, box limits as the upper and lower quartiles. The whiskers extend to 1.5 times the interquartile range and outliers are points outside this range. Specific outliers nominated by OUTRIDER are highlighted in red. **d,** Multiple sequence alignment of several serine/threonine kinases demonstrated that the residues at N345 and D358 in *IRAK1* are highly conserved.

Supplementary Fig. 7

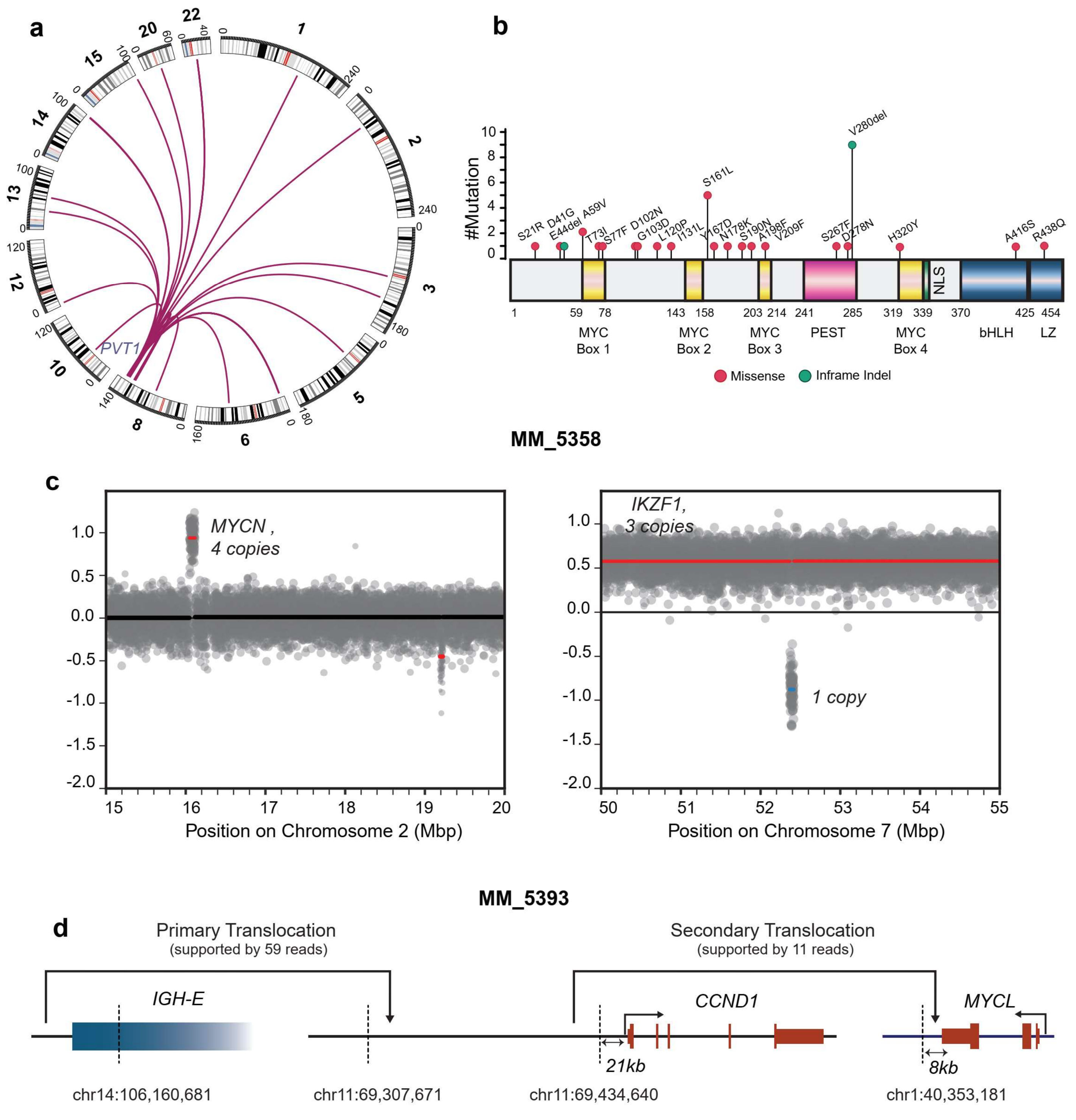

Supplementary Fig. 7 Alterations in the MYC pathway. a, Circos plot shows the fusion partners of *PVT1*. **b,** Lollipop plot for *MYC* mutations aggregated from the RRMM and NDMM cohorts. **c,** An example of *MYCN* rearrangement: a focal, "jumping" amplicon on chromosome 2 containing *MYCN* was inserted into chromosome 7. *MYCN* overexpression was likely driven by the enhancer of the *IKZF1* locus. **d**, An example of a secondary translocation of IgH with *CCND1* and *MYCL* that drove *MYCL* overexpression.

Supplementary Fig. 8

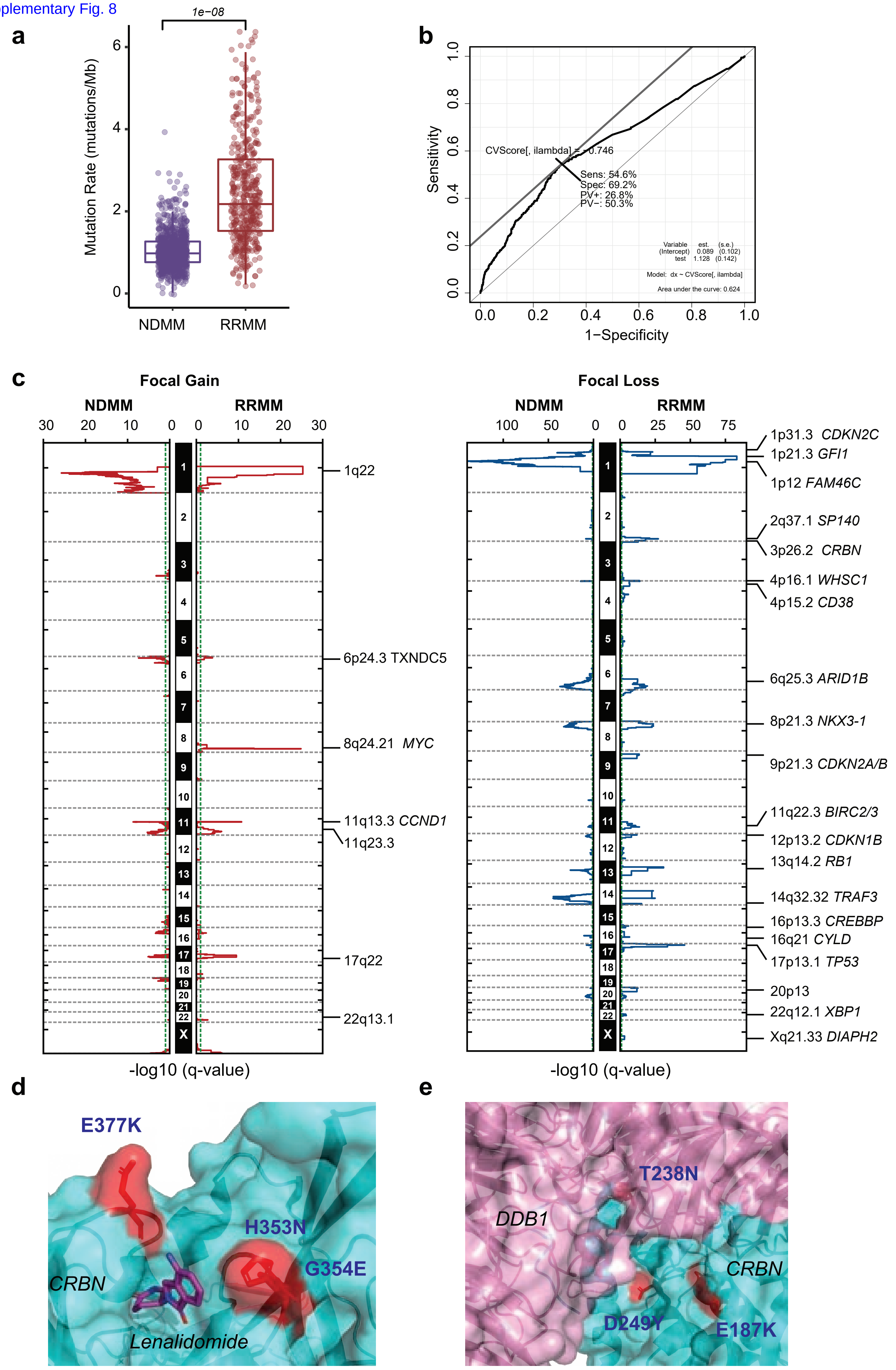

Supplementary Fig. 8 Comparison of mutation and copy-number alterations between relapsed refractory and newly diagnosed MM cohorts. a, Boxplot comparing mutation burden (the number of point mutations per megabase) in the RRMM patients (n=511) relative to NDMM patients (n=965). Box plots include the central line as median, box limits as the upper and lower quartiles, and whiskers extend to 1.5 times the interquartile range. To account for the difference in sequencing depth, only mutations with VAF ≥ 5% were included. P-values were calculated using two-sided Wilcoxon rank-sum test**. b,** Area (AUC) under the Receiver-Operating-Characteristic curve (ROC) of elastic net regularization of the statistical model comparing mutations between two cohorts (Methods). **c,** GISTIC2.0 plot for focal gain and loss in RRMM vs. NDMM. **d,** Mutations in *CRBN* that were near the binding pocket of lenalidomide. **e,** Mutations in *CRBN* at or near the binding interface of *CRBN* and *DDB1* (PDB structure 4CI2[1]).

Supplementary Fig. 9

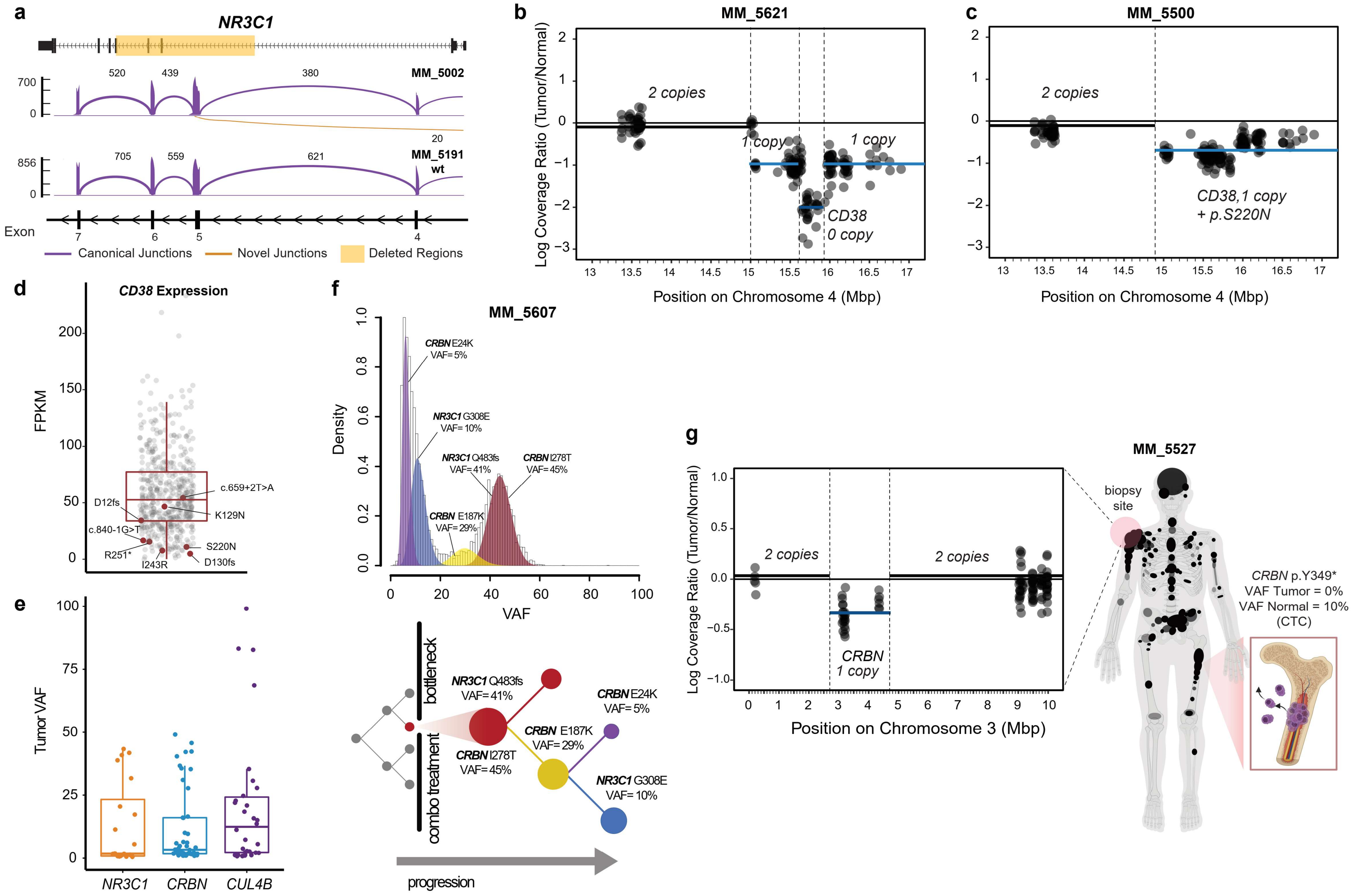

Supplementary Fig. 9 Drug resistance mutations and tumor evolution in RRMM. a, Schematic of an intragenic deletion in *NR3C1*. A de novo splice acceptor downstream of the deletion breakpoint in exon five would cause a de novo splice junction (orange). The new splice product is in-frame. **b,** Example of focal homozygous deletion of *CD38*. **c,** Example of focal hemizygous deletion of *CD38*. The patient also had a point substitution (p.S220N) in the other allele, which may lead to biallelic inactivation of *CD38*. **d,** *CD38* expression (in FPKM) of RRMM patients whose RNA-seq data were available (n=510). *CD38* variants (red) associated with reduced expression of *CD38*. **e,** Variant allelic fraction (VAF) for variants (SNVs and small indels) in *NR3C1* (n= 22), *CRBN* (n=35), and *CUL4B* (n=30). All box plots include the central line as median, box limits as the upper and lower quartiles, and whiskers extend to 1.5 times the interquartile range. **f,** Clonal structure of a case with several drug resistance mutations (MM_5607). Top panel, SciClone was used to perform 1-D clustering of VAF of mutations on diploid regions. Bottom panel, one possible reconstruction of the history of evolution for this tumor. The bottleneck effect was evidenced by the high ("clonal"-like) VAF of *CRBN* and *NR3C1* mutations. Interestingly, the subclones continued to acquire other *CRBN* and *NR3C1* mutations. **g,** Example of inter-clonal heterogeneity: in this case, a *CRBN* stop-gain mutation (Y349*) was only observed in peripheral blood (called from match normal library) but not at the biopsy site. However, a subclonal deletion of CRBN was detected at the biopsy site (called from the tumor library). The illustrations were created using BioRender.com for some elements.

Reference

1. Fischer, E. S. *et al.* Structure of the DDB1-CRBN E3 ubiquitin ligase in complex with thalidomide. *Nature* **512**, 49–53 (2014)
