# Supplementary material for: The genetic heterogeneity and drug resistance mechanisms of relapsed refractory multiple myeloma
Source: Nat Commun. 2022 Jun 29;13:3750. doi: 10.1038/s41467-022-31430-0 (PMC9243087; doi:10.1038/s41467-022-31430-0)
Supplement: Supplementary file 2 — Description of Additional Supplementary Files [file 41467_2022_31430_MOESM2_ESM.pdf]

Description of Additional Supplementary Files

File Name: Supplementary Data 1
Description: Baseline data related to the relapsed refractory multiple myeloma patients sequenced in this study.

File Name: Supplementary Data 2
Description: Mutations identified in the relapsed refractory multiple myeloma cohort.

File Name: Supplementary Data 3
Description: Copy-number alterations and GISTIC2.0 results from the relapsed refractory multiple myeloma cohort.

File Name: Supplementary Data 4
Description: Gene fusions in the relapsed refractory multiple myeloma cohort.

File Name: Supplementary Data 5
Description: Elastic regression results.
If it is convenient for you to mention their name somewhere in the final print, please help us do so. Else, we would be happy with just Supplementary Data 1,2,3, etc., as they are properly explained and referred to in the main text.
