# Supplementary material for: The genetic heterogeneity and drug resistance mechanisms of relapsed refractory multiple myeloma
Source: Nat Commun. 2022 Jun 29;13:3750. doi: 10.1038/s41467-022-31430-0 (PMC9243087; doi:10.1038/s41467-022-31430-0)
Supplement: Supplementary file 8 — Reporting Summary [file 41467_2022_31430_MOESM8_ESM.pdf]

nature research

Corresponding author(s): Arul M. Chinnaiyan
Last updated by author(s): Jun 10, 2022

Reporting Summary

Nature Research wishes to improve the reproducibility of the work that we publish. This form provides structure for consistency and transparency in reporting. For further information on Nature Research policies, see our Editorial Policies and the Editorial Policy Checklist.

Statistics

For all statistical analyses, confirm that the following items are present in the figure legend, table legend, main text, or Methods section.

n/a	Confirmed	
☐	☒	The exact sample size (*n*) for each experimental group/condition, given as a discrete number and unit of measurement
☐	☒	A statement on whether measurements were taken from distinct samples or whether the same sample was measured repeatedly
☐	☒	The statistical test(s) used AND whether they are one- or two-sided<br>*Only common tests should be described solely by name; describe more complex techniques in the Methods section.*
☒	☐	A description of all covariates tested
☐	☒	A description of any assumptions or corrections, such as tests of normality and adjustment for multiple comparisons
☐	☒	A full description of the statistical parameters including central tendency (e.g. means) or other basic estimates (e.g. regression coefficient) AND variation (e.g. standard deviation) or associated estimates of uncertainty (e.g. confidence intervals)
☐	☒	For null hypothesis testing, the test statistic (e.g. F, t, r) with confidence intervals, effect sizes, degrees of freedom and P value noted<br>*Give P values as exact values whenever suitable.*
☒	☐	For Bayesian analysis, information on the choice of priors and Markov chain Monte Carlo settings
☒	☐	For hierarchical and complex designs, identification of the appropriate level for tests and full reporting of outcomes
☒	☐	Estimates of effect sizes (e.g. Cohen's *d*, Pearson's *r*), indicating how they were calculated

Our web collection on statistics for biologists contains articles on many of the points above.

Software and code

Policy information about availability of computer code

Data collection	Paired-end DNA and RNA Sequencing was performed on Illumina HiSeq 2500 instrument. FastQC62 (version 0.11.8) was used to assess read quality per lane. FASTQ conversion was performed with bcl2fastq-1.8.4 in the Illumina CASAVA 1.8 pipeline. Picard (version 2.20.3) was used to monitor other sequencing metrics such as duplication rate, GC biases, and targeted coverage.
Data analysis	For downstream bioinformatics analyses of clinical sequencing data, the following tools were used: Novoalign Multithreaded (version 3.02.08) (Novocraft), Samtools (version 0.1.19), Novosort (version 1.03.02), freebayes (version 1.0.1), pindel (version 0.2.5b9), snpEff (version 4.3t) and snpSift (version 4.3t), bam-readcount (version 0.8.0), sentieon (version 202010.02) (Sentieon, Inc), DNAcopy (version 1.48.0), CNVkit (version 0.9.6) , GISTIC2.0, STAR (version 2.7.4a), featureCounts (version 2.0.0), edgeR (version 3.34.0), oncodriveFML (version 2.0.3), oncodriveCLUSTL (version 1.1.3), MutsigCV8, Mutsig2CV, 20/20+ (version 1.0.1), OUTRIDER (version 1.7.1), PEER, SciClone, with bwa-mem (version 0.7.17), samblaster (version 0.1.25), LUMPY (version 0.3.1), Blast,<br><br>Data visualization and illustration was carried out with Biorender.com and PyMol (version 1.8.2).

For manuscripts utilizing custom algorithms or software that are central to the research but not yet described in published literature, software must be made available to editors and reviewers. We strongly encourage code deposition in a community repository (e.g. GitHub). See the Nature Research guidelines for submitting code & software for further information.

Data

Policy information about availability of data

All manuscripts must include a data availability statement. This statement should provide the following information, where applicable:

- Accession codes, unique identifiers, or web links for publicly available datasets
- A list of figures that have associated raw data
- A description of any restrictions on data availability

All raw sequencing data (fastq files of targeted sequencing and RNA-seq) from RRMM patients enrolled in this study have been deposited in the database of Genotypes and Phenotypes (dbGaP) under accession number phs002498.v1.p1. Raw sequencing data (fastq files of WES and RNA-seq) of the CoMMpass study can be accessed from dbGaP under accession number phs000748.v7.p4. Per dbGaP policy, these datasets are available under controlled access since they contain de-identified individual-level genotype and phenotype information. Principal investigators wishing to access these data must submit their dbGaP Access Applications through the NCBI dbGaP website. Access to these datasets must be renewed annually. Additional information about the application process can be found on dbGaP website. All structures used in the analysis (7DUO and 4CI2) are available on PDB. The remaining data are available within the Article, Supplementary Information, or Source Data file.

Field-specific reporting

Please select the one below that is the best fit for your research. If you are not sure, read the appropriate sections before making your selection.

☒ Life sciences ☐ Behavioural & social sciences ☐ Ecological, evolutionary & environmental sciences

For a reference copy of the document with all sections, see nature.com/documents/nr-reporting-summary-flat.pdf

Life sciences study design

All studies must disclose on these points even when the disclosure is negative.

Sample size	The sample size is not determined by any statistical tests. Sample size (N=511 patients) was determined according to the availability of the clinical samples collected by Molecular Profiling study in the Multiple Myeloma Research Foundation.
Data exclusions	1) Patients who are incarcerated are not eligible to participate.<br>2) Women who are pregnant.<br>3) Patients who have had another malignancy within the last five (5) years (except for basal or squamous cell carcinoma, or in situ cancer of the cervix) where there is a possibility to contaminate the bone marrow aspirate.<br>4) Patients that either had allogeneic stem cell transplants (allo-SCT), or had low tumor purity (<20%) following CD138+ selection were excluded.
Replication	As reported in the figure legends, the findings were reliably reproduced.
Randomization	There is no randomization since this study is not an experimental clinical trial.
Blinding	There is no blinding since this study is not an experimental clinical trial.

Reporting for specific materials, systems and methods

We require information from authors about some types of materials, experimental systems and methods used in many studies. Here, indicate whether each material, system or method listed is relevant to your study. If you are not sure if a list item applies to your research, read the appropriate section before selecting a response.

Materials & experimental systems

n/a	Involved in the study	
☐	☒	Antibodies
☐	☒	Eukaryotic cell lines
☒	☐	Palaeontology and archaeology
☒	☐	Animals and other organisms
☐	☒	Human research participants
☒	☐	Clinical data
☒	☐	Dual use research of concern

Methods

n/a	Involved in the study	
☒	☐	ChIP-seq
☒	☐	Flow cytometry
☒	☐	MRI-based neuroimaging

Antibodies

Antibodies used	Antibodies and their commercial sources are as follows: anti-IL6ST/gp130 (Abcam, ab283685, 1:1,000 dilution), anti-alpha-tubulin (Abcam, ab184577, 1:5,000 dilution), anti-phospho-STAT3-Y705 (Cell Signaling, 9131S, 1:2,000 dilution), and anti-STAT3 (Cell

	Signaling, 4904S, 1:1,000 dilution).
Validation	Information about the validation of these antibodies is available on the manufacturers' websites.

Eukaryotic cell lines

Policy information about cell lines

Cell line source(s)	HEK-293FT was purchased from the ThermoFisher/Invitrogen.
Authentication	This cell line was routinely tested for authentication at the University of Michigan sequencing core with STR profiling.
Mycoplasma contamination	All cell lines in our laboratory are routinely tested for mycoplasma contamination, and cells used in this study are negative for mycoplasma.
Commonly misidentified lines (See ICLAC register)	No commonly misidentified cell lines were used in this study.

Human research participants

Policy information about studies involving human research participants

Population characteristics	Patients must have a diagnosis of multiple myeloma or related malignancy.<br>Patients are undergoing standard of care bone marrow aspirates.<br>Patients (male or female) from any race or ethnicity must be at least 18 years of age at the time of registration.
Recruitment	The recruitment is part of the MMRF Molecular Profiling Protocol (NCT02884102).
Ethics oversight	All samples were acquired after patients provided written informed consent in compliance with the MMRF Institutional Review Board (IRB) (Protocol# MMRF-002; IRB Tracking Number 20151186) and the University of Michigan IRB.

Note that full information on the approval of the study protocol must also be provided in the manuscript.
